# A Genome-Wide Association Study Approach to Identify Novel Major-Effect Quantitative Trait Loci for End-Use Quality Traits in Soft Red Winter Wheat

**DOI:** 10.3390/genes15091177

**Published:** 2024-09-07

**Authors:** Madhav Subedi, John White Bagwell, Benjamin Lopez, Byung-Kee Baik, Md. Ali Babar, Mohamed Mergoum

**Affiliations:** 1 Cornell Institute of Biotechnology, Cornell University, Ithaca, NY 14853, USA; 2 Department of Forestry and Environmental Resources, North Carolina State University, Raleigh, NC 27606, USA; 3 Department of Crop and Soil Sciences, University of Georgia, Griffin Campus, Griffin, GA 30223, USA; 4 Corn, Soybean, and Wheat Quality Research, United States Department of Agriculture, Agricultural Research Service, Wooster, OH 44691, USA; 5 Department of Agronomy, University of Florida, Gainesville, FL 32610, USA; mababar@ufl.edu

**Keywords:** quality, kernel, flour, protein, solvent retention capacity, cookie diameter, softness, genes, quantitative trait loci

## Abstract

Wheat is used for making many food products due to its diverse quality profile among different wheat classes. Since laboratory analysis of these end-use quality traits is costly and time-consuming, genetic dissection of the traits is preferential. This study used a genome-wide association study (GWAS) of ten end-use quality traits, including kernel protein, flour protein, flour yield, softness equivalence, solvent’s retention capacity, cookie diameter, and top-grain, in soft red winter wheat (SRWW) adapted to US southeast. The GWAS included 266 SRWW genotypes that were evaluated in two locations over two years (2020–2022). A total of 27,466 single nucleotide markers were used, and a total of 80 significant marker-trait associations were identified. There were 13 major-effect quantitative trait loci (QTLs) explaining >10% phenotypic variance, out of which, 12 were considered to be novel. Five of the major-effect QTLs were found to be stably expressed across multiple datasets, and four showed associations with multiple traits. Candidate genes were identified for eight of the major-effect QTLs, including genes associated with starch biosynthesis and nutritional homeostasis in plants. These findings increase genetic comprehension of these end-use quality traits and could potentially be used for improving the quality of SRWW.

## 1. Introduction

Various types of wheat classes, each possessing distinct end-use characteristics, are cultivated in diverse environments by communities with different social backgrounds. These wheat classes/varieties are utilized to produce a broad spectrum of end-use products. Therefore, wheat breeding programs worldwide are working on improving grain yield, processing, and end-use quality, with specific breeding targets for each wheat class [1,2,3,4]. For example, soft red winter wheat (SRWW) is bred for low protein content and is suitable for making cookies, crackers, cakes, pretzels, and pastry products [5,6]. The end-use quality of SRWW is determined by milling and baking qualities, which include kernel protein content (KP), kernel hardness, softness equivalence (SE), flour protein content (FP), flour yield (FY), solvent retention capacity (SRC), and cookie diameter (CD).

KP affects the nutritional value, the dough rheological properties, and the baking properties of wheat [7,8]. Unlike SRWW, the other wheat market classes with KP above 12.5% are preferred for bread making [9]. Breeding for higher FY is beneficial, since millers are willing to invest in wheat cultivars that can provide more flour per unit of wheat kernels to increase their revenue. SRC provides an estimation of the flour quality and functionality when the flour is mixed with one of the four different diagnostic solvents: 5% lactic acid (for gluten strength/quality), 5% sodium carbonate (Na_2_CO_3_ for damaged starch), 50% sucrose (for amount of arabinoxylans/pentosans), and water (for water holding capacity) [10]. For soft wheat, the amounts of water, sodium carbonate, and sucrose solutions retained by flour are expected to be low, and the desired level of retained lactic acid solution depends on the type of end-use product [11,12]. Wheat flour of relatively high lactic acid SRC is used for making crackers, as they require strong gluten, whereas wheat flour of relatively low lactic acid SRC is desirable for making cookies and cakes, as they require weak gluten [11,12]. SE provides an estimation of flour particle size and largely depends on kernel hardness. The CD is commonly used as an indicator of SRWW baking quality. The CD and texture are mainly influenced by starch properties, gluten strength, water absorption, and flour texture [13].

Many studies showed that all of the quality-related traits studied are polygenic in nature [14]. Quantitative trait locus (QTL) mapping and genome-wide association studies (GWAS) have been carried out to identify QTLs/genes governing such traits. Simons et al. [15] carried out QTL mapping on recombinant inbred lines (RIL) of spring wheat for twenty end-use quality traits, including six kernel, seven milling and flour, four dough mixing strength, and three bread-making traits, where they identified thirty-one QTLs associated with the traits clustered on five chromosomal regions, 1BS, 1DL, 4BL, 5BL, and 6AS. Cabrera et al. [16] carried out GWAS and QTL mapping for FY, FP, SE, and SRC on a diversity panel and five bi-parental populations, where they identified 26 potential QTL regions in the diversity panel and 74 QTL across all five bi-parental populations. The authors also found high heritability (0.7–0.94) for all of the traits during association analysis. More recently, Gaire et al. [17] carried out GWAS on 270 elite breeding lines of SRWW, where they identified 84 marker-trait associations (MTAs) for seven milling and baking traits, which were grouped into 18 independent QTL regions located on 12 chromosomes of wheat.

However, studies on the end-use quality of wheat are limited, given the number of traits involved and their complex genetic inheritance, specifically for the SRWW [16]. With the use of genomic tools such as GWAS and QTL mapping, novel QTLs/genes related to such traits could be identified and can help in overcoming breeding challenges. The huge genome size of wheat requires an extensive amount of such genetic studies to unfold the mysteries of wheat grain genetics. Therefore, in this GWAS, our goal is to focus on deciphering the genetics of end-use quality traits in SRWW that can contribute towards developing high-yielding wheat cultivars with improved wheat end-use quality. The specific objectives of this study are to (1) evaluate end-use quality traits in a SRWW diversity panel and identify relationships among these traits, and (2) identify novel major-effect QTLs governing these traits using GWAS.

## 2. Materials and Methods

### 2.1. Plant Materials and Experimental Design

A diversity panel of 266 SRWW lines was used to evaluate end-use quality traits. The panel includes advanced lines of SRWW developed by public and private soft wheat breeding programs in the southeastern USA and is referred to as the soft red winter wheat association mapping panel (SWAMP) hereafter [18]. The field trials were conducted at two locations: UGA Southwestern Research and Education Center (SWREC) in Plains, GA (32° 2.80′ N, 84° 21.98′ W), and Bledsoe research farm in Williamson, GA (33° 10.50′ N, 84° 24.46′ W), which are referred to as Plains and Griffin, respectively, in this study. The SWAMP study was evaluated for two consecutive field seasons, 2020–2021 and 2021–2022, in both locations, providing a total of four environment data, Plains-2021, Plains-2022, Griffin-2021, and Griffin-2022. The plot size of the SWAMP experiment was two rows (0.9 m long) with two replicates in Griffin and a plot of seven rows (3 m × 1.5 m) with two replicates in Plains. The experiments were laid out in a randomized complete block design (RCBD) in both locations. The soil was fertilized with pre-plant fertilizer providing Nitrogen, Phosphorous, and Potassium, and planting was carried out in the first three weeks of November at a seed rate of 3 gm m^−1^ using a GPS in-built tractor (John Deere, Moline, Illinois, USA and Trimble technologies, Westminster, CO, USA) and auto trip seed drill (Hege Equipment Inc, Salt lake city, UT, USA). For pre-emergence grass control, Zidua SC was applied at 0.15 L ha^−1^, followed by post-emergence treatments of Harmony Extra at 53 g ha^−1^ for broadleaf control and Axial XL at 1.17 L ha^−1^ for grass control.

### 2.2. Trait Measurement

End-use quality traits were evaluated in the USDA-ARS Soft Wheat Quality Research Unit located in Wooster, Ohio. Seed samples from two replications in each environment were combined to sample 200–400 g of seeds of each line for analysis. The end-use quality traits studied include the following: milling traits: KP, FP, SE, and FY; flour quality: SRC for sodium carbonate (SC.SRC), sucrose (SUC.SRC), lactic acid (LA.SRC), and water (WA.SRC); and baking quality: CD and top-grain (TG). All of the traits except CD and TG were measured as percentages. KP was measured using DA7250 at 12% moisture adjustment. FY was determined using a Quadrumat break roll unit and was calculated as the percentage of total flour weight (Break flour+ mids) obtained from the sample grain weight. FP was estimated as a percentage by using NIR Unity Spectra-Star. SE was the percentage of break flour that passed through the 94-mesh screen of the total flour weight [19]. The SC.SRC, LA.SRC, SUC.SRC, and WA.SRC assays were carried out according to Approved Method 56-11.02 [19]. Sugar-snap cookies were baked according to Approved Method 10-52.2 [19] and CD were measured for two cookies in centimeters. The cookies were graded for a TG score of 1 to 9 based on the islanding pattern on the top surface as described in Approved Method 10-52.2 [19], where 1 is considered poor and 9 is an excellent TG.

### 2.3. Phenotypic Data Analysis

The phenotypic data were analyzed using R v4.3.3 (RStudio, Boston, MA, USA). Three combined datasets were prepared including Griffin-Combined (Griffin-2021, Griffin-2022), Plains-Combined (Plains-2021 and Plains-2022), and All-Combined (Griffin-2021, Griffin-2022, Plains-2021, and Plains-2022). All phenotypic analyses were estimated for the All-Combined dataset. Genotypic and environmental effects were analyzed via analysis of variance (ANOVA). For this, a mixed linear model was used, as shown in Equation (1), where both genotype and environment were considered as random effects.

*Y_ij_* = μ + *G_i_* + *E_j_* + ε_*ij*_ (1)

where *Y*_*i*_*_j_* represents the phenotypic response observed in the j^th^ environment for the i^th^ genotype, μ is the overall mean, *G*_*i*_ is the effect of the i^th^ genotype, *E*_*j*_ is the effect of the j^th^ environment, and ε_*ij*_ represents the residual error term associated with the observation *Y*_*ij*_.

Since there were no replicates, the genotype and environment interaction (GXE) per se could not be evaluated in the datasets [20]. Broad sense heritability was estimated by using the formula as shown in Equation (2):

(2) $$H^{2}=\frac{\sigma_{G}^{2}}{\sigma_{G}^{2}+\sigma_{\frac{e}{n}}^{2}}$$

where H^2^ is the broad-sense heritability estimate; $\sigma_{G}^{2}$ is genetic variance; $\sigma_{e}^{2}$ is residual variance; and n is number of environments.

Pearson correlation was calculated to determine the magnitude and direction of measured trait association in the All-Combined dataset using R package psych v2.3.6 [21] in R. The best linear unbiased prediction (BLUP) was estimated for three combined datasets, Griffin-Combined, Plains-Combined, and All-Combined, using the “lme4” package v1.1 in R [22] and referred to as BLUP-G, BLUP-P, and BLUP-A, respectively.

### 2.4. Genotyping, Linkage Disequilibrium, and Population Structure

The genotyping, marker filtration, linkage disequilibrium, and population structure results have been described previously by Subedi et al. [23]. In short, single nucleotide polymorphisms (SNPs) were filtered for missing values <20% and MAF > 5%, and the obtained number of SNPs was utilized for our analysis. Population-specific linkage disequilibrium critical value was estimated, and any markers exhibiting greater than or equal to this value were regarded as linked and grouped into a common QTL [23]. The number of sub-groups identified through principal component analysis were used as covariates in GWAS.

### 2.5. GWAS Analysis

GWAS using the SWAMP panel was conducted to identify genomic regions significantly associated with end-use quality traits of SRWW using a genome association and prediction integrated tool (GAPIT) v3.0 package in R [24]. GWAS was run using five different models, including both single and multi-loci models: general linear model, mixed linear model, multiple loci mixed model, fixed and random model circulating probability unification (FarmCPU), and Bayesian-information and Linkage-disequilibrium Iteratively Nested Keyway (BLINK). The quantile–quantile (Q-Q) plots and number of significant SNPs identified by each model were compared to select the best model for our analysis [23]. The number of principal components to be used as covariates during GWAS was also confirmed based on model-fit in Q-Q and scree plots.

MTAs with a false-positive discovery rate (FDR) of ≤0.10 were considered to be significant [25]. The adjusted R^2^, which represents the proportion of phenotypic variance (PV) explained, was calculated by modeling the phenotype as a function of the significant marker (independent variable) using the ordinary least-squares regression approach. Pairwise linkage disequilibrium (r^2^) was estimated for significant MTAs identified on the same chromosome using TASSEL v5.0 [26]. Any cluster of markers exhibiting linkage disequilibrium values equal to or superior to the critical r^2^ value was regarded as linked, consequently grouping the markers into a common QTL. Stable loci were defined as any loci, with the associated marker being identified in at least two of the three datasets, BLUP-G, BLUP-P, and BLUP-A.

To explore how the favorable alleles linked to the major-effect QTLs impact the end-use quality traits, we categorized the SWAMP into three groups based on marker genotypes: homozygous for favorable allele, heterozygous, and homozygous for unfavorable allele. These classifications were based on their positive or negative effects on specific traits relative to SRWW. Favorable alleles confer desirable phenotypic characteristics, such as lower FP and FP, higher FY, higher CD, and TG, leading to better end-use quality in SRWW. Subsequently, we conducted TUKEY’s test among these groups using BLUP-A values associated with the trait.

### 2.6. Candidate Gene Discovery and Novelty Testing

Candidate gene discovery was performed by identification of any gene/s lying close to the MTAs. Chinese Spring RefSeq v1.1 reference genome assembly [27] available in Ensembl Plants (http://plants.ensembl.org/, accessed on 3 March 2022) was utilized for the identification of candidate genes for the identified QTLs. Functional annotation of the identified candidate genes was carried out using the Uniport database (https://www.uniprot.org/, accessed on 3 March 2022), Wheat Expression Browser (http://www.wheat-expression.com/, accessed on 3 March 2022), and Wheatomics 1.0 [28]. To test the novelty of the identified QTLs, the locations of these QTLs were compared with the previously identified QTLs using NCBI (https://www.ncbi.nlm.nih.gov/, accessed on 3 March 2022) and PlantBioinfoPF (https://urgi.versailles.inra.fr/, accessed on 3 March 2022) [29] databases.

## 3. Results

### 3.1. Phenotypic Analysis

All the phenotypic data of the studied end-use quality traits is provided in Supplementary Table S1. Our analysis revealed significant variation (*p* < 0.001) among the genotypes of the SWAMP panel for all end-use quality traits in the All-Combined dataset (Supplementary Table S2). The environmental effect was also significant (*p* < 0.001) for all traits. KP ranged from 8.8 to 15.8%, with a mean value of 11.2%. FP also showed wide variation from 6.9 to 14.1%, with a mean of 9.13%. The Griffin environment had higher mean values for KP and FP than the Plains environment (Supplementary Table S2). Interestingly, Plains had slightly higher FY values than the Griffin samples. FY had an overall mean value of 68.9%, ranging from 60.6 to 72.8% (Table 1). SE had a mean value of 58.4%**,** ranging from 40.4 to 70.4%. SRC showed wide variation for all tested solvents, with mean values of 117.5%, 69.7%, 100.7%, and 53.7% for LA.SRC, SC.SRC, SUC.SRC, and WA.SRC, respectively (Table 1). LA.SRC exhibited the highest variation, ranging from 84.9 to 164.9%. For baking traits, CD ranged from 16 to 20.3 cm, while TG scores ranged from 1 to 7.

**Figure 1:**
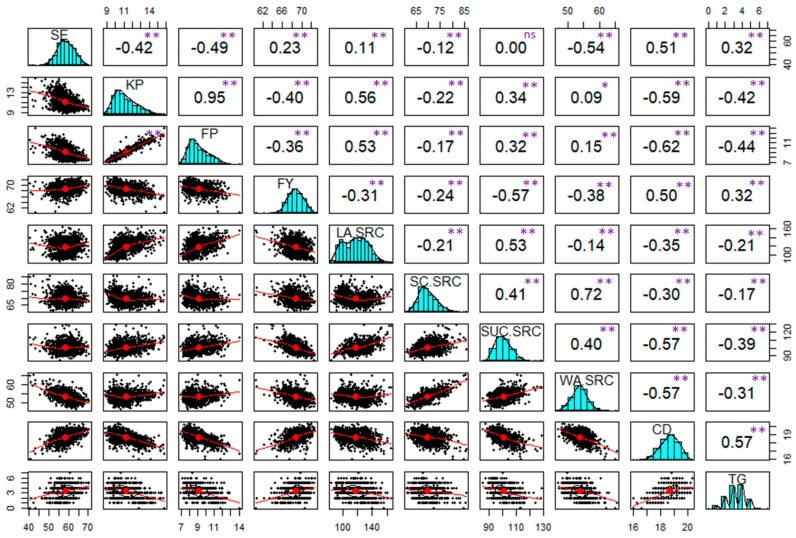
Correlation analysis of ten end-use quality traits among 266 SRWW lines. Combined data from four environments across two years 2020–2022, All-Combined, was used for correlation. SE, softness equivalence; KP, kernel protein; FP, flour protein; FY, flour yield; LA.SRC, lactic acid-solvent retention capacity; SC.SRC, sodium carbonate-solvent retention capacity; SUC.SRC, sucrose-solvent retention capacity; WA.SRC, water-solvent retention capacity; CD, cookie diameter; TG, top-grain; ns, non-significant; * significant at 0.01 level; ** significant at the 0.001 probability level.

Heritability (H^2^) ranged from 0.4 to 0.92 among the end-use quality traits (Table 1). FY, SE, SC.SRC, SUC.SRC, and WA.SRC had H^2^ ≥ 0.9, whereas TG had the lowest heritability of 0.4. The heritability of KP, FP, LA.SRC, and CD were between 0.65 and 0.82. The study revealed significant positive correlations (*p* < 0.01) among all traits except for SE and SUC.SRC (Figure 1). The highest correlation coefficient was observed between KP and FP (r= 0.95). Both KP and FP exhibited positive correlations with LA.SRC (r=0.53 to 0.56), while showing negative correlations with FY (r =−0.40 to −0.36), SE (r = −0.49 to −0.42), and CD (r = −0.62 to −0.59). SE was positively correlated with CD (r = 0.51) and negatively with WA.SRC (r = −0.54). Similarly, FY displayed positive correlations with CD (r = 0.50) and negative correlations with SRC traits (r = −0.57 to −0.24). SC.SRC, SUC.SRC, and WA.SRC were all found to be positively correlated (r= 0.40 to 0.72). Moreover, all SRC traits displayed negative correlations with CD (r = −0.57 to −0.30). Lastly, CD and TG were found to have a positive correlation (r = 0.57).

### 3.2. GWAS

The filtered genotype data, including 27,466 SNPs, is provided in Supplementary Table S3. BLUP-A, BLUP-G, and BLUP-P were used for GWAS. Among the five tested models, mixed linear model, general linear model, multiple loci mixed model, FarmCPU, and BLINK, we found BLINK to have better control over both false-positive and false-negative associations. Thus, BLINK was used to run GWAS for all of the studied end-use quality traits. We identified a total of 80 MTAs at *p*FDR ≤ 0.1 from all three BLUP datasets (Supplementary Table S4). MTAs were identified for all of the traits except CD. Of 80 MTAs, 59 were associated with SRC, including 18 for SC.SRC, 16 for LA.SRC, 15 for WA.SRC, and 10 for SUC.SRC. There were nine MTAs for KP, whereas FP had four MTAs. FY and SE had four and three MTAs, respectively. For baking traits, we only found one MTA associated with TG. Thus, SC.SRC and TG were the traits with the highest and lowest number of MTAs (Supplementary Table S4).

**Figure 2:**
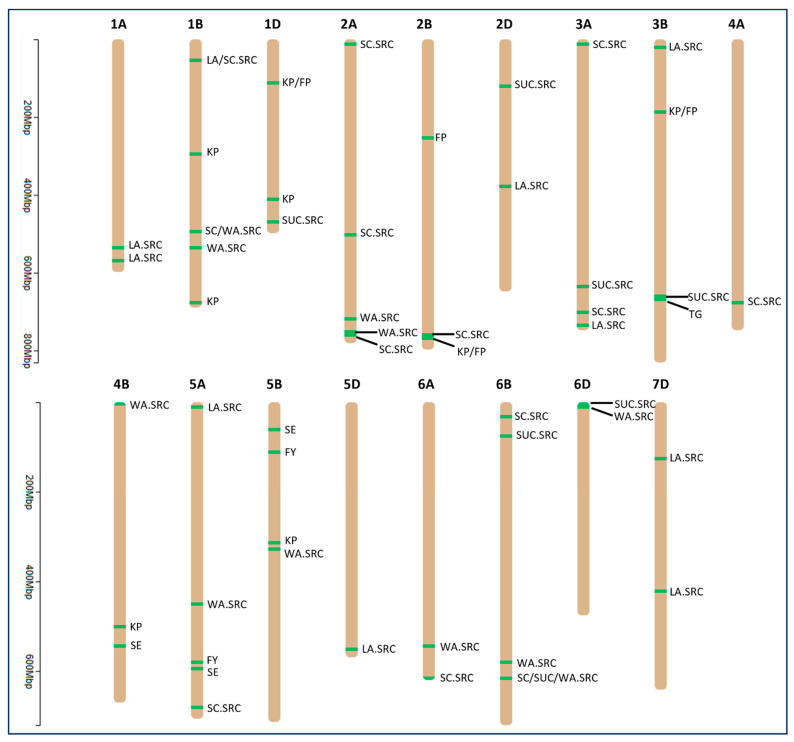
Physical location of MTAs identified for end-use quality traits across 17 chromosomes of wheat genome. The numbers 1 to 7 and the letters A, B, D represent chromosomes in three sub genomes of wheat (A, B, and D). The traits connected with the black lines indicate different MTAs associated with the traits in the chromosomal region. The *y*-axis shows the length of chromosomes in million base pairs (Mbp). SE, softness equivalence; KP, kernel protein; FP, flour protein; FY, flour yield; LA.SRC, lactic acid-solvent retention capacity; SC.SRC, sodium carbonate-solvent retention capacity; SUC.SRC, sucrose-solvent retention capacity; WA.SRC, water-solvent retention capacity; TG, top-grain.

MTAs were discovered across 17 wheat chromosomes, excluding 3D, 4D, 7A, and 7B (Figure 2 and Supplementary Table S5). Chromosome 6B exhibited the highest number of MTAs, totaling 11, while chromosomes 4A and 5D each had only one MTA (Supplementary Table S5). The B genome featured the greatest number of MTAs (38), followed by the A genome (25), with the D genome having the fewest MTAs (17) (Supplementary Table S5).

The marker *S5A_595957121* contributed to the highest percentage of PV explained for SE, reaching 10.3% (Supplementary Table S4). Similarly, marker *S5B_111221616* was linked to the highest PV for FY, amounting to 4.9%. For KP and FP, *S2B_769051134* explained the highest PV, registering 10.9% and 10.3%, respectively. Interestingly, all 13 MTAs related to protein content (KP and FP) were exclusively identified in the B and D genomes (Supplementary Table S4). In contrast, MTAs associated with SRC were identified in 17 chromosomes. For WA.SRC, the marker *S6B_621092809* accounted for the highest PV at 10.9%. Notably, *S1B_55461748* exhibited the highest PV among all of the tested end-use quality traits, contributing to 20.6% in LA.SRC. In the case of SUC.SRC, *S3B_658495375* was associated with the highest PV, explaining 14.5%. For *SC.SRC*, *S6B_619025168* explained the highest PV at 13.9%. Finally, the only marker associated with TG, *S3B_669428408*, explained 12.9% of the PV.

### 3.3. Major Effect QTLs for Milling and Baking Traits

Based on the population-specific linkage disequilibrium critical value of 0.32 [23], we resolved the 80 significant MTAs into 53 distinct QTLs (Supplementary Table S4). Furthermore, we identified 13 QTLs explaining ≥10% PV, which were considered to be major-effect QTLs and are discussed further in Table 2. For example, major-effect QTL, *QFp/Kp.uga-2B*, located on chromosome 2B, explained 10.3% and 10.9% PV for FP and KP, respectively, showing it as a significant locus associated with both traits. Similarly, another major effect, QTL, *QFp/Kp.uga-1D*, located on chromosome 1D, exhibited multi-trait associations contributing to 7.1% and 10.6% of PV for FP and KP, respectively. This investigation also revealed two major-effect QTLs for SE, *QSe.uga-4B* and *QSe.uga-5A*, located on chromosomes 4B and 5A and contributing to 10.1% and 10.3% PV, respectively (Table 2).

We identified eight major-effect QTLs associated with SRC, including two, *QLa/Sc.uga-1B* and *QLa.uga-3A*, on chromosomes 1B and 3A, respectively (Table 2). *QLa/Sc.uga-1B* was associated with two markers in the linkage disequilibrium, *S1B_55461748* and *S1B_65768803*. *QLa/Sc.uga-1B* not only explained 20.6% PV for LA.SRC, but also exhibited a stable association with SC.SRC (up to 9.8% PV) across datasets (BLUP-A and BLUP-P). Furthermore, *QLa.uga-3A*, a stable QTL identified in BLUP-A and BLUP-P, explained up to 11.4% of PV for LA.SRC.

In addition to *QLa/SC.uga-1B*, two more QTLs were identified for SC.SRC, *QSc.uga-6A* and *QSc/Suc/Wa.uga-6B*, positioned on chromosomes 6A and 6B. *QSc.uga-6A*, detected using BLUP-G, contributed to 10.8% PV for SC.SRC (Table 2). QTL *QSc/Suc/Wa.uga-6B* was an interesting finding since this QTL was not only associated with three SRC traits, SC.SRC, SUC.SRC, and WA.SRC, but was also stably expressed for all of these traits among the tested datasets (Table 2). QTL *QSc/Suc/Wa.uga-6B* explained a PV of up to 13.9% for SC.SRC, 14.2% for SUC.SRC, and 11.3% for WA.SRC. Additionally, four more QTLs associated with SUC.SRC were discovered: *QSuc.uga-1D*, *QSuc.uga-2D*, *QSuc.uga-3A*, and *QSuc.uga-3B*, in chromosome 1D, 2D, 3A, and 3B, respectively. *QSuc.uga-2D* and *QSuc.uga-3B* were stable QTLs and explained up to 10.0% and 14.5% of PV, respectively, for SUC.SRC in all tested datasets. In contrast, *QSuc.uga-1D* and *QSuc.uga-3A* were identified in only one dataset, BLUP-A, and explained 11.3% and 12.7% of PV for SUC.SRC. Lastly, we only identified one major-effect QTL for TG, *QTg.uga-3B*, in chromosome 3B, which explained a PV of 12.9% (Table 2).

In summary, among the 13 major-effect QTLs discovered for evaluated end-use quality traits, five exhibited stable expression, and four showed an association with multiple traits (Table 2). Considering the multi-trait association of each QTL, we identified five major-effect QTLs for SUC.SRC, three for SC.SRC, two each for LA.SRC, FP, KP, and SE, and one each for WA.SRC and TG. No major-effect QTLs were identified for FY and CD. These findings underscore the complexity and interrelation of genetic loci governing wheat end-use quality traits.

### 3.4. Allelic Effect of Major-Effect QTLs

We studied the allelic effects for all of the major-effect QTLs except *QTg.uga-3B* (Table 3). There was a significant difference (*p* < 0.05) among the homozygous state of favorable and unfavorable allele genotypes for each of the major-effect QTLs except for *QSe.uga-5A*, with no such difference among the two genotypes. For *QLa.uga-3A*, only one homozygous state was present; therefore, we could not clearly differentiate between favorable and unfavorable alleles (Table 3). In contrast, the difference between homozygous (favorable and unfavorable) and heterozygous genotypes varied with the QTL. *QFp/Kp.uga-1D* exhibited the largest allelic effect for FP and KP, where the lines homozygous for the favorable allele had 0.45% and 0.58% lower FP and KP values compared to lines homozygous for the unfavorable allele (Table 3). Similarly, the lines carrying homozygous favorable alleles associated with *QFp/Kp.uga-2B* had 0.26% and 0.29% lower FP and KP values, respectively, compared to lines with unfavorable alleles. In a similar comparison, for QTL *QSe.uga-4B*, the lines with a favorable allele had a 1.82% lower SE compared to the lines with an unfavorable allele. Notably, for *QSe.uga-5A*, heterozygous lines exhibited 3.57% lower SE values than homozygous lines carrying unfavorable alleles (Table 3).

For *QLa/Sc.uga-1B*, the lines with favorable alleles had 11.2% and 1.48% lower LA.SRC and SC.SRC values, respectively, than the lines with unfavorable alleles (Table 3). In the case of QTL *QLa.uga-3A*, although no lines possessed the homozygous genotype AA, those with the CC genotype had lower LA.SRC values (by 4.10%) than the heterozygous genotype, AC. For *QSc.uga-6A*, the lines with favorable alleles had 4.80% lower SC.SRC values then line with unfavorable alleles. For QTL, *QSc/Suc/Wa.uga-6B*, the lines with favorable alleles had 2.21%, 3.87%, and 1.51% lower SC.SRC, SUC.SRC, and WA.SRC values than the lines with unfavorable alleles. Similarly, for QTLs, *QSuc.uga-2D*, *QSuc.uga-3B*, *QSuc.uga-1D*, and *QSuc.uga-3A* associated with SUC.SRC, the lines with favorable alleles had 11.0%, 10.10%, 4.47%, and 4.78% lower SUC.SRC values than the lines with unfavorable alleles (Table 3).

### 3.5. Candidate Gene Discovery

Out of the 80 significant MTAs identified in this study, 42 were found to be linked to 30 different functional genes (Supplementary Table S4). Based on the annotation results, we identified eight candidate genes for LA.SRC, six for SC.SRC, five for WA.SRC, three for SUC.SRC, six for KP, three for FP, and one each for SE and TG. Candidate genes were identified for eight of the thirteen major-effect QTLs, *QFp/Kp.uga-2B*, *QSe.uga-4B*, *QLa/Sc.uga-1B*, *QSc.uga-6A*, *QSc/Suc/Wa.uga-6B*, *QSuc.uga-1D*, *QSuc.uga-3B*, and *QTg.uga-3B* (Supplementary Table S4, Table 2). However, the proteins associated with candidate genes for *QSe.uga-4B* and *QLa/Sc.uga-1B* have not yet been characterized (Supplementary Table S4).

## 4. Discussion

### 4.1. Variation and Heritability of End-Use Quality Traits

We observed a significant variation in ten end-use quality traits across 266 diverse lines of SRWW. This study showed that KP, FP, SE, FY, WA.SRC, LA.SRC, SUC.SRC, SC.SRC, and CD traits showed high heritability. Our results are in agreement with those reported by Aoun et al. [30], Gaire et al. [17], and Jernigan et al. [20], who showed similar heritability trends for these traits. SRC, FY, and SE, in particular, have been considered highly heritable, repeatable, and thus reliable for the evaluation of soft wheat quality in breeding stocks [14,31]. This implies that these trait values are mostly influenced by genetic factors. Therefore, this can be a useful result for implementing genetic selection for these traits in breeding programs to improve the end-use quality of wheat.

### 4.2. Relationship among End-Use Quality Traits

The correlation values allow us to understand the relationships among various end-use quality traits. We can leverage these correlations to access various milling, flour, and baking qualities of wheat by analyzing a few properties/traits that show a strong correlation with traits of interest. For instance, CD is a very important indicator of the baking quality of soft wheat. However, we need a large amount of flour for baking cookies, and it requires a lot of time. Instead, CD can be predicted through correlated traits such as FP, WA.SRC, SUC.SRC, and SE [32,33], which are much easier to measure and require fewer resources. We also found that all four traits, FP, WA.SRC, SUC.SRC, and SE, have a higher correlation with CD than other traits. Thus, this study provides additional support in recommending these traits for accessing CD.

We also found a significant correlation between WA.SRC, SC.SRC, and SUC.SRC, similar to the results from Gaire et al. [17]. This correlation is reasonable, since all three of the traits are positively correlated with damaged starch level and influence the water absorption/holding capacity of the flour [32]. Our study also showed a significant negative correlation between SE and KP. Large values of SE indicate soft grain texture [34]. Therefore, soft wheat is much higher in SE but lower in KP than hard wheat [34]. A similar significant negative correlation was also reported between FP and FY, which could be due to the differences in the amount of energy plants require to produce protein and carbohydrate molecules [6,35].

Moreover, FP and KP showed the highest positive correlation in the SRWW panel, which was similar to results for SRWW from Aoun et al. [36]. This is expected, since most of the KP is stored in the endosperm [37]. We also found a negative correlation of SE with KP and WA.SRC. However, the correlation between SE and SUC.SRC was not significant. Gaire et al. [17] also reported no correlation between these traits in their SRWW panel. Additionally, KP and FP exhibited strong positive correlations with LA.SRC in alignment with the result from Gong et al. [38]. The correlation between protein content and LA.SRC was expected, as the latter is known to be determined by the former and protein strength [10].

### 4.3. Major-Effect QTLs for End-Use Quality Traits

We identified 80 significant MTAs located on 17 different chromosomes of wheat that were associated with all of the studied end-use quality traits except CD. PV explained by these MTAs ranged from 0.1 to 20.6%. Thirteen major-effect QTLs were identified, which explained ≥ 10% PV. We found several major-effect QTLs associated with two or more of the correlated traits. For instance, major-effect QTLs *QFp/Kp.uga-1D* and *QFp/Kp.uga-2B* were associated with both KP and FP, *QSc/Suc/Wa.uga-6B* was associated with SC.SRC, SUC.SRC, and WA.SRC, and *QLa/Sc.uga-1B* with LA.SRC and SC.SRC traits. Recent studies [17,30] also reported similar multi-trait associations of loci with the same and/or different end-use quality traits. These QTLs can be a useful tool for the selection or simultaneous improvement of these traits in wheat breeding programs.

Stable QTLs are invaluable in breeding programs, as the introgression of such QTLs allows breeders to develop improved and adapted varieties. This also allows us to study the adaptation mechanism of plants to varying environmental conditions. We found five stable and major-effect QTLs in this study, *QLa/Sc.uga-1B*, *QLa.uga-3A*, *QSc/Suc/Wa.uga-6B*, *QSuc.uga-2D*, and *QSuc.uga-3B*, all of which are QTLs associated with SRC traits. *QLa/Sc.uga-1B* and *QSc/Suc/Wa.uga-6B* are of particular interest to us since these QTLs are not only stable, but also show associations with multiple SRC traits, as mentioned earlier. Moreover, these QTLs were also responsible for explaining the highest PV for all of the tested SRC traits among all of the major-effect QTLs. For instance, *QLa/Sc.uga-1B* explained 20.6% of PV for LA.SRC. Similarly, *QSc/Suc/Wa.uga-6B* explained 13.9%, 14.2%, and 11.3% of PV for SC.SRC, SUC.SRC, and WA.SRC. As such, these two inherently stable and multi-traits associated QTLs exhibit substantial potential for the concurrent enhancement of wheat end-use traits while preserving its stability across diverse environmental conditions.

One of the objectives of our study was to identify novel QTLs associated with end-use quality traits. Previously, several studies have identified genomic regions associated with the end-use quality traits on the same chromosomes identified in this study. For instance, the *Glu-D1* gene has been identified on chromosome 1D [39]. Liu et al. [40] identified QTLs associated with KP on chromosome 1D and 2B, namely *QGpc.cd1-1D.1*, *QGpc.cd1-1D.2*, *QGpc.cd1-1D.3*, *QGpc.cd1-1D.4*, *QGpc.cd1-2B.1*, and *QGpc.cd1-2B.2*. Similarly, Gaire et al. [17] identified MTA related to SE on chromosome 4B. Moreover, QTLs associated with various SRC traits have been identified [17,20,30] on chromosomes 1B, 3A, 6A, 1D, and 3A, similar to our findings in this study. However, none of the reported MTAs/QTLs from these studies overlap with the major-effect QTLs identified in this study, except for QTL *QTL-SC.uga.6A*. Jernigan et al. [20] identified a QTL for FP at the 612 Mb region of chromosome 6A, associated with marker Excalibur_rep_c98042_438, which is close and possibly the same QTL to our SC QTL *QTL-SC.uga.6A* at 611Mb. Thus, the other 12 major-effect QTLs identified in this investigation are considered to be putative novel loci associated with these end-use quality traits. These results could be attributed to the limited genetic studies addressing end-use quality traits of wheat compared to other traits such as agro-morphological traits. In addition, there is a challenge of identifying the physical locations of the markers associated with the QTLs from studies conducted before the reference genome was established. This is particularly the case when these markers that have not been defined in common databases, such as GrainGenes (https://wheat.pw.usda.gov/GG3/), that are used by researchers for such novelty testing.

Identification of candidate genes helps us narrow down our QTLs to a few genes, some of which govern our traits of interest. However, the identification of such genes itself requires an extensive amount of study. Here, we speculate on the candidate genes solely based on the proximity of the genes to our QTLs under a small window of the reference genome. Among our 13 major-effect QTLs, we found candidate genes for eight of them. Also, proteins produced by some of these genes were found to be associated with some aspects of end-use quality determination in plants. For instance, *TraesCS6A02G404700*, which is a candidate gene for QTL *QSc.uga-6A* that was associated with SC.SRC, has been reported to play a significant role in starch accumulation and biosynthesis [41,42]. As for SC.SRC, an indicator of starch damage in wheat, the candidate gene seems to be a potential causative gene for the QTL. Additionally, *TraesCS6B02G353300*, the candidate gene for QTL *QSc/Suc/Wa.uga-6B* associated with SC.SRC, SUC.SRC, and WA.SRC was annotated to produce a metal tolerance protein that plays an important role in maintaining homeostasis of nutrients in plants [43,44], thus making it potentially associated with the end-use quality of wheat.

### 4.4. Genetic Complexity of End-Use Quality Traits and Breeding Implications of Identified QTLs

Most of the MTAs identified in this study had small effects, and few major-effect QTLs were detected per trait. This hints towards the genetic and genomic complexity of end-use quality traits and the selective breeding effects of these traits in soft red winter wheat breeding programs. End-use quality traits are quantitative traits governed by many genes with small effects, as evidenced in our MTA findings. Previous studies [17,30] similarly detected mostly minor-effect QTLs for various end-use quality traits.

The relatively modest size of our diversity panel (230 lines used for GWAS) may have potentially limited our ability to detect major-effect QTLs, as observed in prior research [16]. Additionally, although the D genome plays a significant role in determining wheat quality attributes, its relative genetic homogeneity compared to the A and B genomes may have further reduced polymorphisms for detecting QTLs located in the D genome [45,46,47]. Notably, our SWAMP panel also had fewer SNPs and MTAs within the D genome than both the A and B genomes.

Among the major-effect QTLs, the favorable alleles of seven of the major-effect QTLs were in high frequency, reflecting continuous high selection pressure for these alleles in SRWW breeding programs. Aoun et al. [30] reported such frequency of favorable alleles in soft white wheat and speculated them to be the result of long-term phenotyping, selection, and pyramiding of favorable alleles across breeding populations. As the favorable alleles become increasingly fixed within breeding populations, the polymorphism associated with the QTL becomes harder to detect and is often filtered out to improve the quality of genotyping data. This fixation of major alleles may further explain the high heritability obtained for most of the traits [30].

Given that the SWAMP panel utilized in this GWAS comprises elite lines adapted to the US southeast, the lines possessing favorable alleles for the major-effect QTLs can be utilized directly in regional breeding programs. Molecular marker technologies, such as Kompetitive Allele-Specific PCR (KASP), can be developed for these QTLs to facilitate the selection of progenies carrying these desirable alleles across successive breeding generations. This marker-assisted selection approach for end-use quality traits not only conserves resources, but also reduces the time required for laboratory analyses [6]. Moreover, breeders can augment their breeding strategies by integrating genomic selection methods, which enable the simultaneous utilization of both major- and minor-effect QTLs to predict the end-use quality of wheat lines with greater accuracy and efficiency.

## 5. Conclusions

In summary, we conducted a GWAS to investigate ten end-use quality traits in a diversity panel of 266 SRWW lines that showed significant variation for all evaluated traits. Most of these traits showed high heritability, indicating significant genetic control of these traits. In total, 27,466 SNPs were used for GWAS, and QTLs associated with the traits were identified across 17 chromosomes of wheat. Thirteen major-effect QTLs were identified, twelve of which were deemed to be putative novel loci. Candidate genes were also identified for these QTLs, some of which were associated with certain aspects of wheat quality. Overall, these results, upon further validation, can be a useful resource for researchers and breeders to improve the end-use quality of wheat through the development of molecular markers associated with the major-effect QTLs and using them in marker-assisted/genomic selections in wheat improvement programs. However, further studies to validate these findings under different environmental conditions are warranted.

## Figures and Tables

**Table 1:** Descriptive statistics, analysis of variance, and heritability estimation for ten end-use quality traits studied in 266 SRWW lines.

Trait ^a^	General Statistics of Population						H^2 g^
	Mean	SD ^b^	CV ^c^	Min ^d^	Max ^e^	SE ^f^	
KP (%)	11.2	0.56	4.95	8.8	15.8	0.03	0.65
FY (%)	68.6	1.38	2.01	60.6	72.8	0.09	0.90
SE (%)	58.4	3.05	5.22	40.4	70.4	0.19	0.92
FP (%)	9.1	0.49	5.42	6.9	14.1	0.03	0.72
LA.SRC (%)	117.5	8.91	7.58	84.9	164.9	0.55	0.81
SC.SRC (%)	69.7	2.91	4.17	61.1	85.7	0.18	0.91
SUC.SRC (%)	100.7	5.26	5.23	84.9	128.7	0.33	0.90
WA.SRC (%)	53.7	1.88	3.51	46.7	65.5	0.12	0.90
CD (cm)	18.6	0.42	2.23	16.0	20.3	0.03	0.82
TG	3.3	0.6	17.68	1	7	0.04	0.40

^a^ End-use quality traits KP, kernel protein; FY, flour yield; SE, softness equivalence; FP, flour protein; LA.SRC, lactic acid solvent retention capacity; SC.SRC, sodium carbonate solvent retention capacity; SUC.SRC, sucrose solvent retention capacity; WA.SRC, water solvent retention capacity; CD, cookie diameter; TG, top-grain. ^b^ SD, standard deviation. ^c^ CV, coefficient of variation. ^d^ Minimum value obtained for traits. ^e^ Maximum value obtained for traits. ^f^ SE, standard error of the mean. ^g^ Broad-sense heritability estimates for the traits from the All-Combined dataset.

**Table 2:** Summary of 13 major-effect QTLs for ten end-use quality traits from three BLUP datasets, BLUP-G, BLUP-P, and BLUP-A.

QTL Name ^a^	SNP ^b^	Trait ^c^	Dataset ^d^	Allele ^e^	MAF ^f^	*p*FDR ^g^	Effect ^h^	PV ^i^	Candidate Gene ID ^j^	Associated Protein ^k^
*QFp/Kp.uga-1D*	S1D_108803007	FP	BLUP_A	G/A	0.2	0.04	−0.15	7.15	-	-
	S1D_108803007	KP	BLUP_A	G/A		0.00	−0.18	10.67		
*QFp/Kp.uga-2B*	S2B_769051134	FP	BLUP_A	A/G	0.3	0.00	0.13	10.36	*TraesCS2B02G581400*	Leucine-rich repeat-containing N-terminal plant-type domain-containing protein
		KP	BLUP_A			0.00	0.14	10.92	*TraesCS2B01G581500*	Pentatricopeptide repeat-containing protein
*QSe.uga-4B*	S4B_544593051	SE	BLUP_P	T/C	0.4	0.00	−0.81	10.12	*TraesCS4B02G269500*	Uncharacterized protein
*QSe.uga-5A*	S5A_595957121	SE	BLUP_P	G/A	0.1	0.01	1.62	10.30	*-*	-
*QLa/Sc.uga-1B*	S1B_55461748	LA.SRC	BLUP_G	T/C	0.1	0.00	5.64	20.64	*TraesCS1B02G070400*	Uncharacterized protein
	S1B_65768803	SC.SRC	BLUP_A		0.2	0.00	−0.97	9.49	*TraesCS1B02G082300*	Uncharacterized protein
		SC.SRC	BLUP_P			0.00	−0.96	9.81		
*QLa.uga-3A*	S3A_738748059	LA.SRC	BLUP_A	C/A	0.1	0.00	7.87	11.41	*-*	-
		LA.SRC	BLUP_P			0.00	3.41	10.93		
*QSc.uga-6A*	S6A_611293571	SC.SRC	BLUP_G	C/T	0.1	0.03	1.37	10.83	*TraesCS6A02G404700*	Serine/threonine-protein phosphatase
*QSc/Suc/Wa.uga-6B*	S6B_619025168	SC.SRC	BLUP_A	A/C	0.3	0.00	−1.36	13.98	*TraesCS6B02G353300*	Cation efflux protein cytoplasmic domain-containing protein/Metal tolerance protein
		SC.SRC	BLUP_P			0.00	−1.30	13.85		
		SUC.SRC	BLUP_A			0.01	−1.46	12.43		
		SUC.SRC	BLUP_P			0.00	−1.63	14.27		
	S6B_621092809	SC.SRC	BLUP_G	T/G	0.3	0.00	1.15	11.44		
		WA.SRC	BLUP_A			0.00	0.73	11.33		
		WA.SRC	BLUP_G			0.00	0.61	10.99		
*QSuc.uga-1D*	S1D_462736410	SUC.SRC	BLUP_A	C/T	0.2	0.01	−1.67	11.34	*TraesCS1D02G393000*	Dynamin-related protein 5A
*QSuc.uga-2D*	S2D_121596645	SUC.SRC	BLUP_A	A/G	0.1	0.01	2.48	10.01	*-*	-
		SUC.SRC	BLUP_P			0.00	3.14	8.95		
*QSuc.uga-3A*	S3A_635786446	SUC.SRC	BLUP_A	T/G	0.1	0.01	−1.82	12.79		
*QSuc.uga-3B*	S3B_658495375	SUC.SRC	BLUP_A	A/G	0.1	0.01	2.12	14.55	*TraesCS3B02G421500*	Jasmonate O-methyltransferase
		SUC.SRC	BLUP_P		0.1	0.01	2.36	14.47	*TraesCS3B01G421600*	Transcription initiation factor TFIID subunit 9
*QTg.uga-3B*	S3B_669428408	TG	BLUP_P	C/T	0.2	0.00	−0.08	12.96	*TraesCS3B02G429900*	Formin-like protein

^a^ Major-effect QTL identified in this study. ^b^ SNP associated with major-effect QTL. ^c^ End-use quality traits. SE, softness equivalence; KP, kernel protein; FP, flour protein; FY, flour yield; LA.SRC, lactic acid-solvent retention capacity; SC.SRC, sodium carbonate-solvent retention capacity; SUC.SRC, sucrose-solvent retention capacity; WA.SRC, water-solvent retention capacity; TG, top-grain. ^d^ BLUP datasets used for identification of the marker-trait association. BLUP-A, BLUP values from All-Combined dataset; BLUP-P, BLUP values from Plains-Combined dataset; BLUP-G, BLUP values from Griffin-Combined dataset. ^e^ Allele combination for the locus. ^f^ MAF, minor allele frequency. ^g^ *p*FDR, False discovery rate adjusted *p*-value. The markers are significant at less than 0.1 *p*FDR. ^h^ Allelic effect on the trait. In the GAPIT-based GWAS result, the effect is estimated for the second marker in alphabetical order, which means it could be a minor or major allele effect. ^i^ Percentage of phenotypic variance (PV) explained by the marker. ^j^ Candidate genes identified for the QTL. ^k^ Protein produced by the candidate gene.

**Table 3:** Allelic effect of significant markers associated with major-effect QTLs identified in this study.

QTL Name ^a^	SNP ^b^	Traits ^c^	MAF ^d^	Genotype ^e^	N ^f^	Mean BLUP-A ^g^	TUKEY HSD Test ^h^
*QFp/Kp.uga-1D*	S1D_108803007	FP	0.20	AA	6	9.54	A
				AG	67	9.25	B
				GG	145	9.09	C
		KP	0.20	AA	6	11.75	A
				AG	67	11.37	B
				GG	145	11.17	C
*QFp/Kp.uga-2B*	S2B_769051134	FP	0.34	AA	131	9.06	A
				AG	37	9.18	AB
				GG	60	9.32	B
		KP	0.34	AA	131	11.15	A
				AG	37	11.28	AB
				GG	60	11.44	B
*QSe.uga-4B*	S4B_544593051	SE	0.43	CC	85	59.34	A
				CT	24	58.47	AB
				TT	116	57.52	B
*QSe.uga-5A*	S5A_595957121	SE	0.09	AA	33	60.09	-
				AG	1	56.52	A
				GG	192	58.65	-
*QLa/Sc.uga-1B*	S1B_55461748	LA.SRC	0.14	CC	16	107.9	A
				CT	27	115.66	B
				TT	183	119.1	C
	S1B_65768803	SC.SRC	0.14	CC	36	70.74	A
				CT	25	70.6	B
				TT	165	69.26	C
*QLa.uga-3A*	S3A_738748059	LA.SRC	0.14	CC	200	117.08	A
				AC	26	121.18	B
				AA	0	NA	-
*QSc.uga-6A*	S6A_611293571	SC.SRC	0.08	CC	202	69.56	A
				CT	9	69.62	A
				TT	9	74.36	B
*QSc/Suc/Wa.uga-6B*	S6B_619025168	SC.SRC	0.26	AA	160	70.37	A
				AC	17	68.67	B
				CC	47	68.16	B
		SUC.SRC		AA	160	101.77	A
				AC	17	100.86	AB
				CC	47	97.9	B
	S6B_621092809	WA.SRC	0.26	GG	45	52.59	A
				GT	22	53.48	AB
				TT	157	54.1	B
*QSuc.uga-1D*	S1D_462736410	SUC.SRC	0.20	CC	171	101.61	A
				CT	8	98.77	AB
				TT	32	97.14	B
*QSuc.uga-2D*	S2D_121596645	SUC.SRC	0.07	AA	200	100.45	A
				AG	15	101.45	A
				GG	4	111.45	B
*QSuc.uga-3A*	S3A_635786446	SUC.SRC	0.11	GG	17	104.89	A
				GT	5	103.35	AB
				TT	197	100.11	B
*QSuc.uga-3B*	S3B_658495375	SUC.SRC	0.15	AA	166	99.88	A
				AG	53	103.31	B
				GG	3	109.98	B

^a^ Major-effect QTL identified for end-use quality traits. ^b^ Significant SNPs associated with major-effect QTLs. ^c^ End-use quality traits for which the MTAs were identified. SE, softness equivalence; KP, kernel protein; FP, flour protein; FY, flour yield; LA.SRC, lactic acid-solvent retention capacity; SC.SRC, sodium carbonate-solvent retention capacity; SUC.SRC, sucrose-solvent retention capacity; WA.SRC, water-solvent retention capacity; TG, top-grain. ^d^ MAF, minor allele frequency. ^e^ Homozygous and heterozygous genotypes for each SNP. The allele in bold is the favorable allele for the trait. ^f^ N, number of wheat lines having respective genotype classes for each marker. ^g^ Mean value of traits in BLUP-A. ^h^ Tukey’s HSD test results, where different letters represent significant differences at *p* < 0.05.

## Data Availability

The GBS SRA data generated for this panel can be accessed at NCBI using accession number PRJNA578088 (https://www.ncbi.nlm.nih.gov//bioproject/PRJNA578088, accessed on 3 March 2022).

## Supplementary Materials

The following supporting information can be downloaded at: https://www.mdpi.com/article/10.3390/genes15091177/s1. Table S1: Phenotypic data of 266 wheat lines characterized for ten end-use quality traits in four individual environments; Table S2: Descriptive statistics and analysis of variance results for studied end-use quality traits on 266 advanced soft red winter wheat lines; Table S3: Genotypic data of SRWW lines used in the study; Table S4: 80 MTAs identified in the study that were resolved into 53 QTLs and Table S5: Total number of MTAs per chromosome.
